# Enrichment of Agar Gel with Antioxidant Pectin from Fireweed: Mechanical and Rheological Properties, Simulated Digestibility, and Oral Processing

**DOI:** 10.3390/gels8110708

**Published:** 2022-11-02

**Authors:** Sergey Popov, Vasily Smirnov, Nikita Paderin, Daria Khramova, Elizaveta Chistiakova, Fedor Vityazev, Victoria Golovchenko

**Affiliations:** Institute of Physiology of Federal Research Centre “Komi Science Centre of the Urals Branch of the Russian Academy of Sciences”, 50 Pervomaiskaya Str., 167982 Syktyvkar, Russia

**Keywords:** agar, pectin, hydrogel, phenolic compounds, texture, rheology, simulated digestion, electromyography, acceptability

## Abstract

The aims of the study were to evaluate the influence of pectin isolated from fireweed (FP) on the mechanical and rheological properties of agar (A) gel, to investigate the release of phenolic compounds (PCs) and pectin from A-FP gels at simulated digestion in vitro, and to evaluate the oral processing and sensory properties of A-FP gels. The hardness of A-FP gels decreased gradually with the increase in the concentration of FP added (0.1, 0.4, and 1.6%). The hardness of A-FP1.6 gel was 41% lower than A gel. Rheological tests found A gel was a strong physical gel (storage modulus (G′) >>loss modulus (G″)), and the addition of FP up to 1.6% did not significantly change its G’. The G″ value decreased in A-FP gels compared to A gel. The release of galacturonic acid (GalA) was 3.4 ± 0.5, 0.5 ± 0.2, 2.4 ± 1.0, and 2.2 ± 0.7 mg/mL after digestion of A-FP1.6 gel in the oral in vivo phase (OP) and subsequent incubation in simulated gastric (SGF), intestinal (SIF), and colonic (SCF) fluids in vitro. The incubation medium after OP, SGF, and SIF digestion of A-FP1.6 contained 24–64 μg GAE/mL of PCs, while SCF contained 144 μg GAE/mL, supposing a predominant release of antioxidant activity from the gel in the colon. Chewing to readiness for swallowing A-FP gel required less time and fewer chews with less activity of the masseter and temporalis muscles. A-FP1.6 gel had a lower likeness score for taste and consistency and a similar score for appearance and aroma when compared with A gel. Thus, A-FP gels were weakened compared to A gel and required less time and muscle activity for oral processing. A-FP gel had antioxidant activity due to the PCs associated with pectin, while A gel had no antioxidant activity.

## 1. Introduction

A combination of hydrocolloids is often used to modify rheological characteristics and impart novel mouthfeels to food gels. Agars and pectins are important hydrocolloids obtained by extraction of marine red seaweed and higher terrestrial plants, respectively [1]. Agar is a polysaccharide in which residues of 3,6-anhydro-L-galactose and D-galactose are linked in a linear chain. Upon heating, the galactan chains take a random and rigid coil conformation, form helices, and form a gel network of combined thick bundles upon subsequent cooling [2]. Thermo-reversible agar gelation does not require additional cross-linkers, such as cations, and is convenient for use in the culinary, food, and confectionery industries [3]. The high polymerization of blocks formed by two different polysaccharides, alpha and beta bonds, makes it difficult for agar to be cleaved by carbohydrate enzymes [4]. Agar is a dietary fiber, since the human digestive system does not produce agarase, and agar can be partially fermented and metabolized when entering the large intestine, which is populated by bacteria [5,6]. Therefore, agar has a prebiotic effect, stimulating the growth of Lactobacillus spp. and Bifidobacterium spp. and increasing the production of short-chain fatty acids [7,8]. In addition, agar has a convenient effect as a bulking and laxative agent because of its gelling capacity, and it has been found to possess anti-tumor, anti-oxidative, and hypoglycemic effects [9].

Pectins are complex anionic polysaccharides that can be isolated from various plant materials. Pectins used in the food industry are mainly obtained from citrus peels and apple pomace [10]. The pectin backbone comprises 1,4-linked α-D-galacturonic acid (GalA) residues, with partly methyl- and/or acetyl-esterified carboxyl groups. Side chains composed of neutral monosaccharides (arabinose and galactose residues, predominantly) linked to rhamnose residues inserted in the galacturonic backbone, forming branched areas of the pectin macromolecule [11]. Pectin with low (<50%) and high (>50%) degrees of methyl-esterification are classified as belonging to the low- (LMP) and high-methyl-esterified (HMP) types, respectively [12]. HMP gels at low pH in the presence of sugar form a physical network. Multivalent cross-linking cations are required for gelation of LMP, which can occur over a wide pH range [13]. However, the use of sugar in large quantities may limit the use of pectin gels in a healthy diet, while the use of various cross-linkers may impair the palatability of the pectin gel. The high biological activity makes it promising to include pectin into complex food gels. In particular, pectin has been shown to possess antioxidant activity [14,15,16,17,18]. Which functional groups of the pectin macromolecule mediate the antioxidant activity remains unknown. However, some authors believe that the antioxidant activity is due not to polysaccharide chains but to the phenolic compounds (PCs) associated with them [19].

LMP possessing antioxidant activity higher than commercial apple pectin has previously isolated from leaves of fireweed (Epilobium angustifolium L.) [20]. Fireweed pectin (FP) fractions scavenge 1,1-diphenyl-2-picrylhydrazyl- and superoxide radicals in vitro depending on the content of associated PCs [20]. In the present study, we assumed that incorporating FP into the agar gel would make it possible to obtain a mixed food gel with antioxidant activity. It has previously been shown that incorporating other hydrocolloids into agar gel changes its physicochemical properties [21,22,23]. Rozek et al. [24] supplemented model agar gel with grape PCs. However, the features of agar-pectin gel and enrichment of agar gel with PCs associated with polysaccharide have not been previously studied.

The aim of the study was to evaluate the influence of FP on the mechanical and rheological properties of agar gel and to investigate the release of PCs and pectin from A-FP enriched gels at simulated digestion in vitro. Electromyography (EMG)-measured oral processing and sensory properties of A-FP gels were also evaluated with the involvement of volunteers.

## 2. Results

### 2.1. General Characterization and Mechanical Properties of Gels

Agar gels enriched with antioxidant pectin were prepared by heating/cooling a mixture of 1.5% agar and 0.1, 0.4, or 1.6% FP and named A-FP0.1, A-FP0.4, or A-FP1.6, respectively. Total PC content in A-FP0.1, A-FP0.4 and A-FP1.6 gels was 23 ± 5, 84 ± 17, and 418 ± 101 μg gallic acid equivalents (GAE)/g, respectively (Table 1).

Tg and Tm represent the gelling and melting temperatures, respectively; PCs, phenolic compounds; GAE, gallic acid equivalents; WL, the weight-loss during drying. SD, standard deviation.

**Figure 1:**
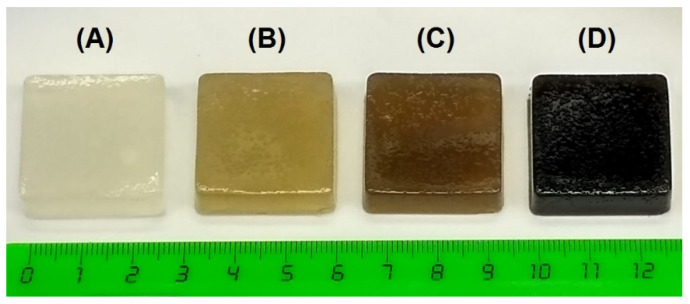
Appearance of A (**A**), A-FP0.1 (**B**), A-FP0.4 (**C**), and A-FP1.6 (**D**) gels.

The images of 1.5% agar gel (A) and A-FP mixed gels are shown in Figure 1. Evidently, A gel had a light transparent structure in comparison with A-FP gels. The color of the gel became markedly darker depending on the concentration of added FP. The dark color of pectin-containing gels is, apparently, because of the presence of PCs in FP.

**Figure 2:**
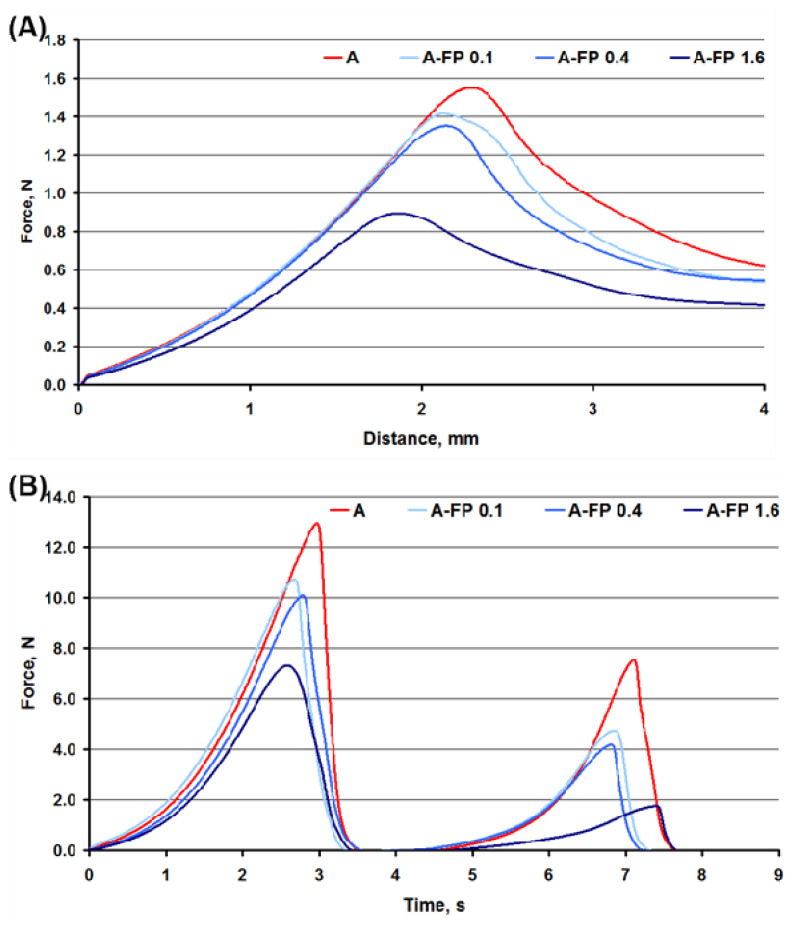
Mean (*n* = 12) force over distance (**A**) and over time (**B**) curves in puncture test and double compression tests, respectively, of A and A-FP gels.

The mechanical behavior of A and A-FP gels was first monitored in the puncture test, and all gels demonstrated force–distance curves with one peak (Figure 2A). Hardness, determined as a peak force through the puncture of A-FP0.1, A-FP0.4, and A-FP1.6 gels was 9, 14, and 44% lower than that of A gel, respectively (Table 1). The consistency of A-FP0.1, A-FP0.4, and A-FP1.6 gel was 13, 16, and 37% lower than that of A gel, respectively.

The texture profile analysis (TPA) results are presented in Figure 2B and Table 2. FP decreased the hardness of A gel. The hardness of A-FP1.6 gel was 41% lower than A gel. Similar data was obtained for cohesiveness and chewiness. The cohesiveness of A-FP0.1, A-FP0.4, and A-FP1.6 gel was 1.3, 1.6, and 2.2 times lower than that of A gel, respectively. The chewiness of A-FP0.4 and A-FP1.6 gels was 1.5 and 3 times lower, respectively, than that of A gel. However, A-FP1.6 gel exhibited the highest levels of springiness and resilience. The springiness and resilience of A-FP1.6 gel was 1.2 and 2.8-fold of A gel sample, respectively.

### 2.2. Rheological Properties of Gels

**Figure 3:**
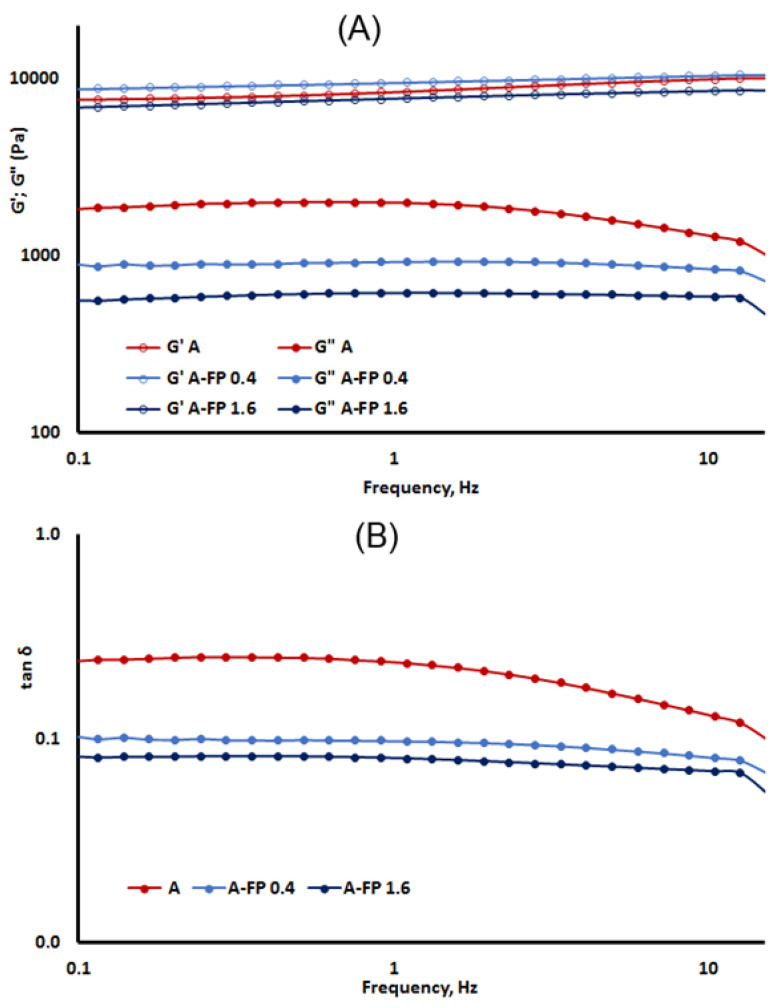
Storage modulus (G’, void symbols) and loss modulus (G”, filled symbols) test (**A**) and tan δ test results (**B**) represented as a function of frequency.

The viscoelastic properties of A and A-FP gels were studied using oscillatory assessments with recording of the parameters of storage or elastic modulus (G’) and the loss or viscous parameters modulus (G”). Angular frequency sweep measurements are conducted to estimate the structural integrity and gel strength. The mechanical spectra are given in Figure 3A.

Both A and A-FP gels demonstrated high G’ values (10,000 > G’ > 8500 Pa) when the modulus had only a slight dependence on frequency. The loss factor tan δ << 1 confirmed the solid state of A and A-FP gels (Figure 3B). The same results were obtained for the A-FP0.1 gel (data not shown). Thus, A gel was a strong physical gel, and the addition of FP up to 1.6% did not significantly change its G’, since the G’ values for all studied gels were the same (Figure 3A). The G” value decreased in A-FP gels compared to A gel (Figure 3A), coinciding with the viscosity values given in Table 3.

**Figure 4:**
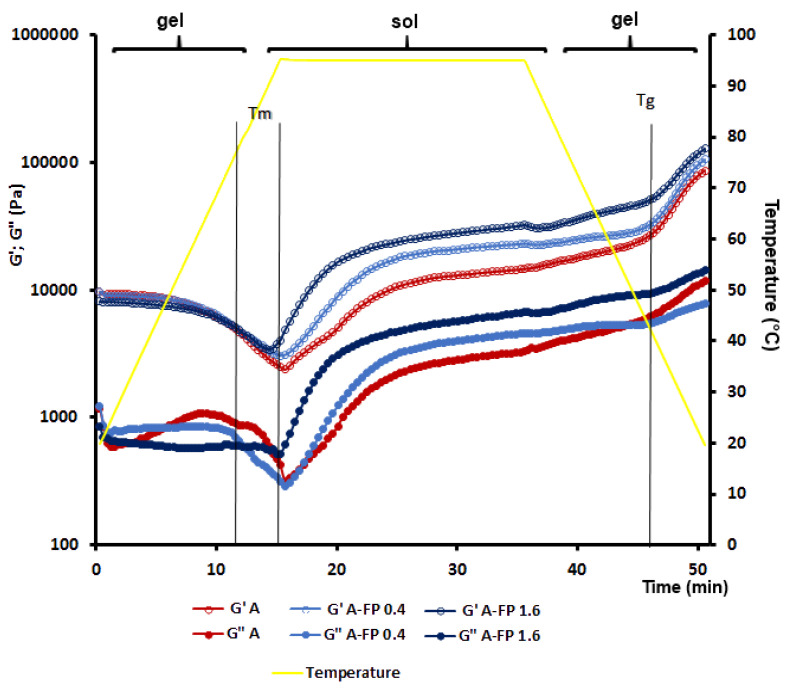
Temperature ramp tests performed at constant frequency (1.0 Hz) for A, A-FP0.4, and A-FP1.6 gels. An initial heating step (5 °C/min from 20 to 95 °C) was followed by an isothermal step (95 °C, 20 min) and a final cooling step (rate: 5 °C/min from 95 to 20 °C).

The temperature sweep experiments showed that the G’ values of A and A-FP gels first decreased upon heating to 69–71 °C, and then increased (Figure 4). FP failed to significantly affect the melting temperature (Tm) of agar gel (Table 1). The G’ values of A-FP gels exceeded that of A gel during the isothermal (95 °C) step. All samples demonstrated sharply increased G’ values upon cooling below 41 °C, indicating a sol–gel transition. The gelling temperature (Tg) of A-FP1.6 was slightly (0.2 °C) higher than that of A gel, as presented in Table 1. The G” values of A gel were higher than A-FP gel at the melting step (Figure 4). However, after the phase transition, the G” values of A sol were lower than A-FP sol during the isothermal step.

### 2.3. Pectin and PCs Release during Simulated Digestion of Gels

**Figure 5:**
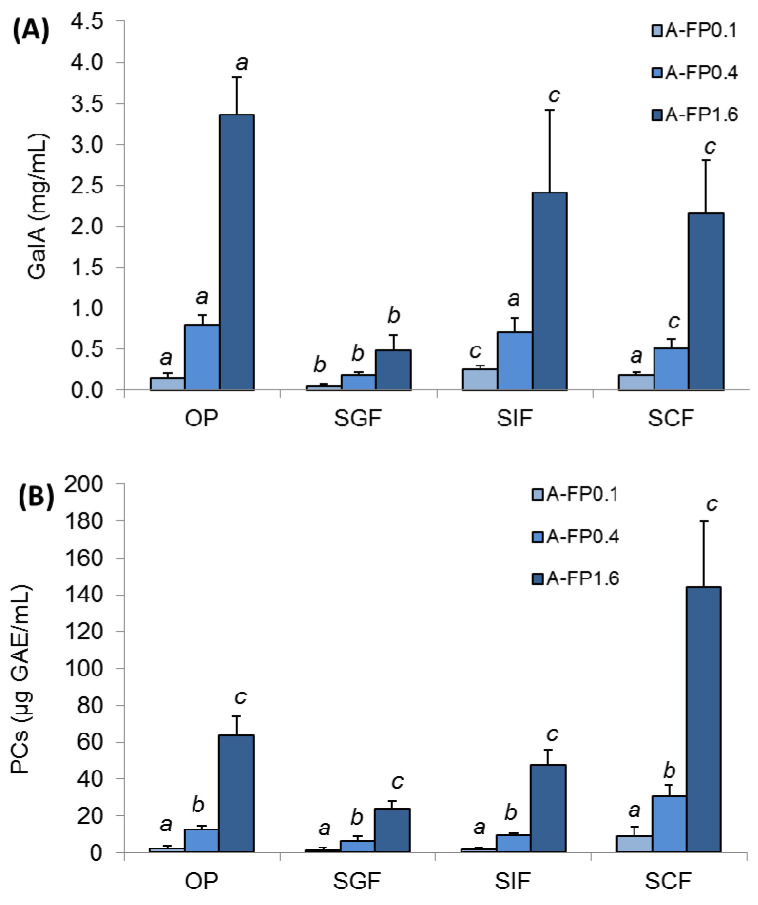
The amount of galacturonic acid (GalA) residues (**A**) and total phenolic compounds (PCs) (**B**) released from A-FP gels during successive oral in vivo (OP) and gastrointestinal in vitro phases of digestion. SGF, SIF, and SCF—simulated gastric, intestinal, and colonic fluid, respectively. Mean ± SD. Different letters among means for the same gel in different phases of digestion—*p* < 0.05 (*n* = 6).

The incubation medium after each phase of simulated digestion of A-FP gels contained polysaccharide fractions. The molecular weight of polysaccharides was 225 ± 16, 154 ± 30, 216 ± 11, and 119 ± 24 kDa after successive oral in vivo (OP) and gastrointestinal in vitro digestion of A-FP1.6 in simulated gastric (SGF), intestinal (SIF), and colonic (SCF) fluids, respectively. The concentration of GalA in the incubation medium was measured to determine the release of pectin chains from the agar gel upon digestion since GalA is a major component of FP. The amount of released GalA increased in proportion to the increase in the amount of incorporated pectin at each individual phase of digestion (Figure 5A). When comparing different phases of digestion, A-FP gel released the highest level of GalA at the chewing in vivo (OP). The release of GalA from A-FP0.1, A-FP0.4 and A-FP1.6 into SGF was minimal, namely 3.0, 4.4 and 6.9 times less, respectively, than during OP. The release of GalA in SIF and SCF then increased significantly compared to SGF, but was less than during OP (Figure 5A). The incubation medium of the digestion of A gel did not contain high molecular polysaccharides or GalA (data not shown).

PCs released from A-FP gels were evaluated during successive OP and SGF, SIF, and SCF digestion to predict the part of the gastrointestinal tract where FP would have an antioxidant effect. The incubation medium after OP, SGF, and SIF digestion of A-FP1.6 contained 24–64 μg GAE/mL of PCs, while SCF contained 144 μg GAE/mL Figure 5B.

### 2.4. Oral Processing of Gels

**Figure 6:**
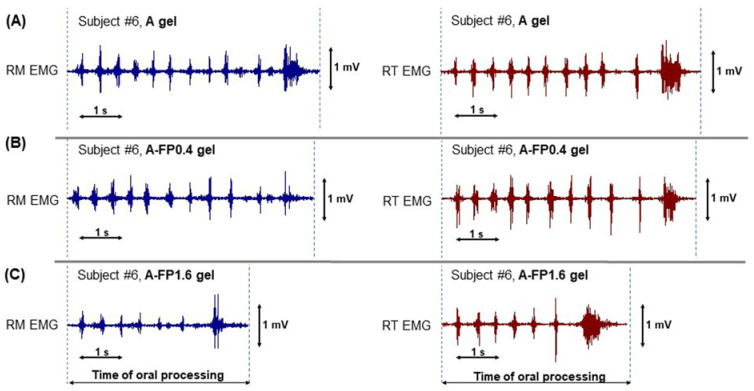
Example of electromyography (EMG) during chewing A (**A**), A-FP0.4 (**B**), and A-FP1.6 (**C**) gels in a representative subject. EMG signals of right masseter (RM) and right temporalis (RT) muscles are recorded. Area between the dotted lines indicates the time of oral processing.

Oral processing of A and A-FP gels was investigated using EMG with a unilateral chewing style. All participants freely chewed gel samples on the preferred chewing side of the jaw (left or right) in their habitual manner and did not report any difficulty in performing the task. Typical EMG signals from rhythmic masseter and temporalis activities during chewing of A, A-FP0.4, and A-FP1.6 gels are shown in Figure 6. Oral processing of A-FP0.1 gel was not studied because of the low amount of FP incorporated and released.

Chewing to readiness for swallowing A-FP1.6 gel required 30% less time and 27% fewer chews (Figure 6C, Table 4). Consistent with this, masseter and temporalis activity were approximately 30% lower when chewing A-FP1.6 then when chewing A gel. A-FP0.4 failed to change the chewing pattern compared to A gel (Table 4). Despite the shortening of the chewing time over the sequence, the duration of the chewing cycle failed to change significantly. The chewing cycle lasted, on average, 700 ms for both A and A-FP gels (data not shown). The chewing frequency was similar in all treatments and was equal to 1.5 s^−1^ (data not shown).

### 2.5. Acceptability of Gels

**Figure 7:**
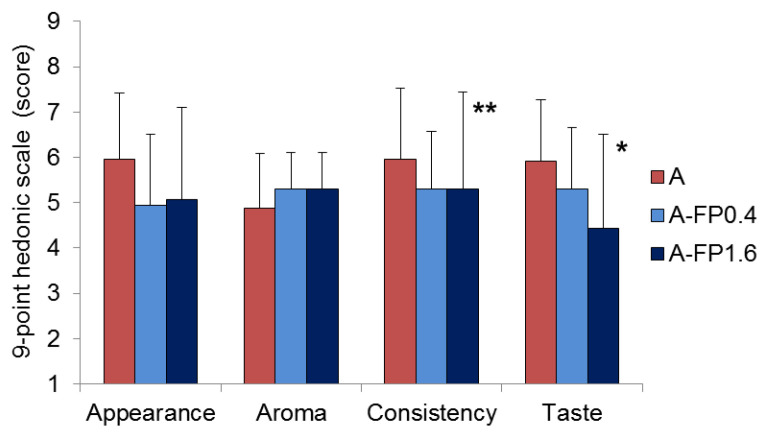
Sensory scores of A, A-FP0.4, and A-FP1.6 gels. Data are presented as the mean rating (n = 16). * and ** *p* < 0.05 and 0.01 vs. A gel.

Likeness-scores of A, A-FP0.4, and A-FP1.6 gel samples are displayed in Figure 7. A-FP1.6 gel had a lower likeness-scores for taste and consistency and similar scores for appearance and aroma when compared with A gel. The acceptability of A-FP0.4 gel did not differ from that of A gel.

## 3. Discussion

The results demonstrated that FP was efficiently incorporated into A gel by the method used. The efficiency of loading of pectin-associated PCs into agar gel was calculated to be equal to 78–97% as the total phenolic content in FP was earlier determined to be as much as 27 mg GAE/g [20].

**Figure 8:**
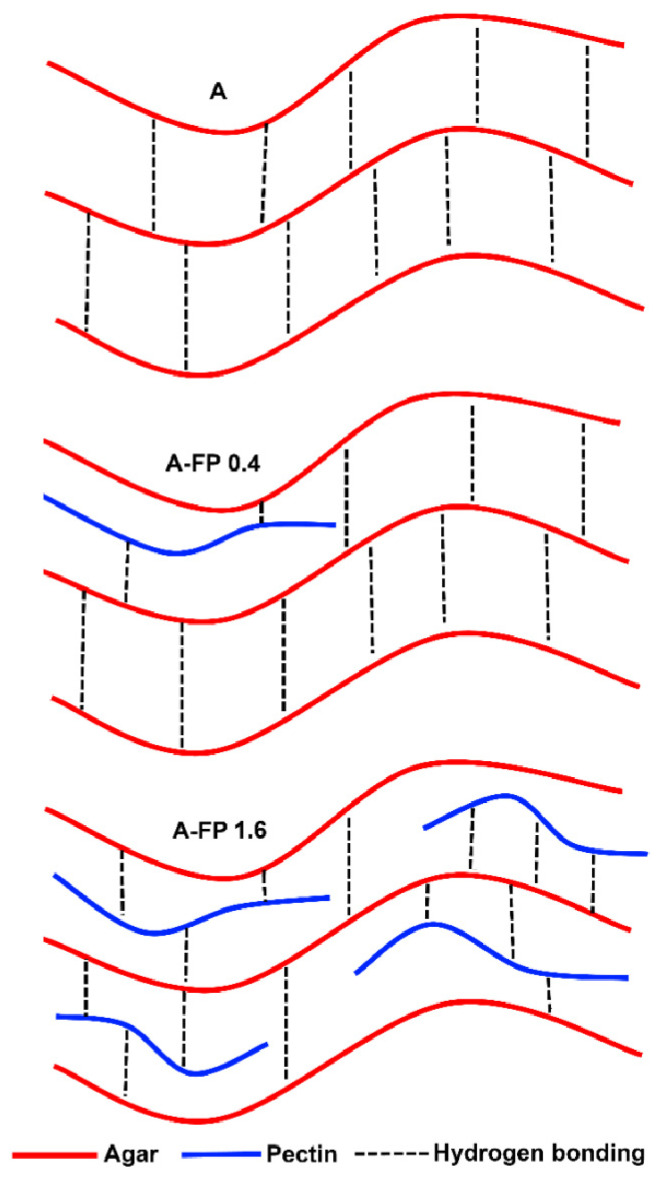
Schematic illustration of a proposed gel network in A and A-FP gels.

The mechanical behavior observed under puncture and double compression showed that the structure of A-FP gels was weakened compared to A gel. Hydrogen bonding is well known to be involved in agarose gelation [3]. The decrease in hardness, which reflects the maximum load applied to the specimens, the consistency, which represents the required work of deformation, and the cohesiveness, which reflects the stability of the internal structure, suggests that pectin molecules interfere with the formation of hydrogen bonds between agar molecules (Figure 8). The formation of hydrogen bonds with the hydroxyl groups of pectin can decrease the density of hydrogen bonds between the adjacent D-galactose and 3,6-anhydro-L-galactose residues of the agarose chains.

The decrease in the mechanical strength of A-FP gels may also result from the decrease in pH caused by the addition of FP to agar gel. The decrease in A gel strength by FP is consistent with a previous report [25], where the authors imply carrot juice is able to weaken agar gel because of pectin content. Incorporating other polysaccharides may reduce the mechanical properties of the agar gel system. Locust bean gum (LBG), a galactomannan from the seeds of the carob tree, and salep polysaccharide, a glucomannan from the roots and tubers of orchids, have been shown to reduce strength and elongation of agar film [21]. In another study [22], the addition of a combination of LBG and xanthan gum reduced the breaking stress of the agar gel membrane. However, other studies have reported that LBG may increase the strength of agar gels [23]. According to a rheological study, FP seemed to dissolve in the aqueous phase of rigid A gel since A and A-FP gels are not significantly different in G’ value. This result was expected, as FP, being an LM pectin, needs divalent cations to form a gel network. The addition of FP reduced the G” value of A gel, which is similar to the effect of konjac gum on agar gel [26].

The sequential digestion model was used to predict the bioactivity of the A-FP gel in different parts of the digestive tract. It is known that the ability of pectins to bind glucose and lipids in the small intestine determines their hypoglycemic and hypolipidemic action [27,28], whereas in the large intestine, pectins stimulate beneficial microflora [29]. Chewing released a large portion of the pectin from the A-FP gel during its destruction in the oral cavity. However, the pectin is then released predominantly in SIF and SCF, thus showing a great potential for bioactivity. The minimal release of pectin in the SGF is likely because of the low pH, which promotes an excessive aggregation of polysaccharide helices. The measurement of the reactivity of incubation medium with the Folin–Ciocalteu’s reagent is related to the ability to donate electrons, and therefore indicates antioxidant activity. The preferential release of PCs in the SCF indicates that digesting A-FP gel will keep its antioxidant potential until the colonic environment.

The textural and rheological properties of food gels determine their mechanical behavior during mastification, including chewing, bolus formation, and swallowing [30]. Furthermore, hardness, cohesiveness, chewiness, and springiness are critical parameters for the sensory perception of food. Hardness is considered an important indicator for the consumer, which can simulate the force required for food to compress between teeth or between tongue and palate. Cohesiveness is the extent to which a material can deform before it breaks, simulating the compression of food before teeth chew it in the mouth. Chewiness refers to the energy required for chewing solid food in a swallowable state [31]. The EMG results of the chewing of A-FP gel are consistent with the data of texture analysis. Softening A gel with FP resulted in chewing it easier has made chewing it easier. The results of EMG measuring the chewing of A-FP gel are consistent with the data of texture analysis. Softening A gel with FP made it easier to chew. Various studies have demonstrated that consumers adapt their chewing pattern to the textural properties of foods [30,32]. The observation that the harder samples required increased muscle activity for the oral processing has been reported for agar caramels [33], gellan [34] and gelatin [35] model gels.

A weaker and faster chewable A-FP gel may be required by children who prefer soft products [36], the elderly who have difficulty chewing hard foods [37], and patients of dysphagia [38]. However, the softening of A gel because of the addition of FP lowered its acceptability. In disagreement with these data, [39] and [40] reported that softer agar jelly or yogurt, respectively, with softer agar particles have higher liking scores than harder samples. In addition, overall acceptability of pectin-containing jams is higher in softer samples [41,42]. It is assumed that decreasing hardness enhances the flavor intensity, which could lead to an increase in liking of samples. In the present study, the reduced weight-loss during drying (WL) and lower serum release from A-FP1.6 gel may explain its poorer acceptability properties, because the separation of liquid from the gel network affects the perceived texture and taste [43]. It should also be taken into account that a shorter chewing time of A-FP gel can be associated with fewer signals from the oral cavity reaching the brain. In addition, shorter chewing may decrease saliva production which is important for taste perception [32]. However, saliva production was not determined in our study. In any case, the lower taste and consistency ratings of A-FP gel require further improvement in gel formulation to improve its acceptability for consumers.

## 4. Materials and Methods

### 4.1. Materials

Agar (A) powder was purchased from Zhenpai Hydrocolloids Co., Ltd. (Zhangzhou City, Fujian Province, China), with moisture 7.97 wt%, pH 6.46, and 1.5% gel strength of 1150 g/cm^2^. FP was isolated from the leaves of *E. angustifolium* L. by the treatment of plant raw materials with aqueous hydrochloric acid at pH 0.8 [20]. The PC content in FP was 27 mg GAE/g. The chemical characteristics of the polysaccharides are shown in Table 5.

The reagents, including the Folin and Ciocalteu’s phenol reagent and pectinase from *Aspergillus niger* (1.18 U/mg), were purchased from Sigma-Aldrich (St. Louis, MO, USA). Gallic acid was purchased from MP Biomedicals (Solon, OH, USA). Ethanol (95%, JSC Kirov Pharmaceutical Factory, Kirov, Russia), pyridine, boric acid, toluene and chloroform stabilized with ethyl alcohol (Vekton, Saint Petersburg, Russia), methanol (Merck, München, Germany, 99.5%), 3,5-dimethylphenol (Sigma Aldrich, USA, 99%), sodium borohydride (Sigma Aldrich, USA, 98.5%), Sulphuric acid (Sigma-tec, Moscow, Russia) were used.

### 4.2. Preparation and General Characterization of A and A-FP Gels

A (1.5 g), FP (0.1, 0.4, or 1.6 g), and sugar (5.0 g) were dispersed in deionized water (up to 100 g) and left for one hour under magnetic stirring. The dispersion was heated to 95 °C in a slow cooker for 45 min. The hot solution was vigorously shaken and transferred into a silicone form (10 × 26 × 26 mm), followed by cooling to room temperature for 2 h. The gels were covered with a film and kept at 4 °C before being used for further experiments.

The pH was determined using an InLab^®^ Science Pro-ISM pH electrode and an S20 SevenEasy™ pH meter (Mettler-Toledo AG, Schwerzenbach, Switzerland) on homogenates of A and A-FP gels. Gel was weighed and mechanically broken in distilled water in a ratio of 1:10 (*w*/*v*).

WL was determined as a measure of the water-holding capacity of the gels. The weight of 12 gel samples batch before and after complete removal of moisture by drying samples was determined by AG 245 weight (Mettler Toledo International) WL was calculated as:

WL % = (WW − WD)/WW × 100 %,(1)

where WW and WD represent the weight of the gel samples before and after drying, respectively.

Serum release was determined using the TA-XT Plus Texture Analyzer (Texture Technologies Corp., Stable Micro Systems, Godalming, UK). A pre-weighed dry filter paper was placed under the gel block to absorb the serum being exuded from the gel. The gels were compressed twice to 80% of initials their height at room temperature. The pre- and post-test speed was 5.0 mm/s and the test speed was 1 mm/s. The wet filter paper was weighed immediately after the compression. Serum release was calculated by the equation: (weight of the released serum)/(initial weight of gel). Twelve replicates were made of each type of gel.

For measurement of PC content, the gel sample (~4 g) was weighed and mechanically broken. After adding water in a ratio of 1:4 (*w*/*v*) the mix was heated in a water bath for 10 min and cooled to room temperature. The content of PCs was determined in the resulting solution with the Folin–Ciocalteu reagent using gallic acid as a standard [44]. The results were expressed as μg of gallic acid equivalents (GAE) per mL of solution or as a percentage of the total amount of released PCs and remaining in the residue.

The PC loading was calculated using the following equation:

Loading PCs (μg GAE/g gel) = (A/B) × 100,(2)

where A and B are the PC content (μg GAE/mL) and the concentration of gel in solution (g/mL), respectively. The encapsulate efficiency was calculated using the following equation:

Loading efficiency (%) = (A/B) × 100,(3)

where A and B are the measured and theoretical amounts of PCs (in μg GAE/g gel), respectively. The theoretical amounts of PCs in A-FP gel were calculated according to the total PC content in FP.

### 4.3. Measurement of Mechanical Properties

For the puncture test, gel samples (9 mm height, 14 mm length and 14 mm width) were placed on the platform of the texture analyzer (Texture Technologies Corp., Stable Micro Systems, Godalming, UK). The test was performed using a cylindrical aluminum probe P/5 (5 mm in diameter). The method settings, including the pretest, test, and post-test speeds, were 5.0, 1.0, and 5.0 mm/s, respectively. The distance (depth of insertion) was set at 4 mm of the initial height of the samples. Several mechanical parameters were extrapolated from the force–distance curves: puncture hardness (the peak force that occurs during the puncture), elasticity (distance at the peak force), and consistency (the area of work during the puncture). The test was performed at room temperature. Each analysis was executed 12 times.

For the two-cycle compression test, gel samples (9 mm height, 14 mm length and 14 mm width) were placed on the platform of the texture analyzer (Texture Technologies Corp., Stable Micro Systems, Godalming, UK). The test was performed using a cylindrical aluminum probe P/25 (25 mm in diameter). The gels were compressed twice at room temperature. The pre- and post-test speed was 5.0 mm/s, and the test speed was 1 mm/s until a 100% strain. A destructive 100% strain was used to represent gels behavior during the chewing process. Twelve replicates were made of each type of gel. For two cycles, compression–decompression provided a force–time graph and led to the extraction of eight parameters: hardness, cohesiveness, springiness, gumminess, chewiness, and resilience. All calculations were performed using Texture Exponent 6.1.4.0 software (Stable Micro Systems, UK) with manufacturer recommendations.

### 4.4. Measurement of Rheological Properties

The rheological properties of the samples were determined in a rotational-type rheometer (Anton Paar, Physica MCR 302, Graz, Austria) equipped with a parallel plate geometry (25 mm in diameter) and a gap of 1–4 mm between the two plates. Four repetitions were performed for each sample.

Temperature sweeps were carried out from 95 to 5 °C at a rate of 5.0 °C/min. The storage modulus (G’) and loss modulus (G”) values were recorded by a temperature sweep test at a constant stress of 9.0 Pa and an angular frequency of 1.0 Hz (linear viscoelastic region). Tg was determined as the temperature when G’ and G” crossed over [45,46].

The frequency sweeps were expressed in terms of G’ and G” through an angular frequency range of 0.01–10 Hz. The tests were performed at 20 °C and a constant stress of 9.0 Pa, which was within the linear viscoelastic region [47]. The degree of frequency dependence for the G’ was determined using the power-law parameters [48,49], expressed as:

G’ = A × 𝜔 × B(4)

where G’ is the storage modulus, 𝜔 is the oscillation frequency (Hz), and A is a constant.

### 4.5. In Vivo Oral Phase (OP) and Static In Vitro Gastrointestinal Digestion

OP digestion was performed as proposed previously [43]. Each type of gel (~4 g) was chewed 20 times by six healthy people (3 men and 3 women) to simulate the maximum destruction of the sample in the oral cavity according to preliminary testing in which the largest number of chews was 20. Thereafter, the bolus was spat three times into a beaker to reduce sample loss. Immediately after this, 4.0 mL of water was added to the beaker, mixed by shaking, and all the liquid part was separated for analysis. The gel pieces were transferred to a 20 mL jacketed glass vessel (reactor) for further in vitro digestion.

In vitro gastrointestinal digestion was approached by the method [50] with minor modifications. After OP, gels were sequentially incubated in 4.0 mL of the pre-heated SGF (pH 1.5, 0.08 M HCl, and 0.03 M NaCl), SIF (pH 6.8, 0.05 M KH_2_PO_4_, and 0.02 M NaOH), and SCF (0.01 M KH_2_PO_4_, 0.05 M NaHPO_4_, and pectinase: 1.7 mg/mL) at 37 °C and under continuous orbital shaking (250 rpm) for 2, 4, and 18 h, respectively. Before replacing it with another, the medium was completely separated from the gel by a grid (mesh size 350 μm) and used for analysis. After incubation in SCF, the gel residue was destroyed by heating (95 °C, 10 min) in water (16 mL). The data from SCF and gel residue were summed.

The contents of GalA and PCs were determined in fluid after each (OP, SGF, SIF, and SCF) phase of digestion. For this, aliquots (1–2 mL) of incubation medium were taken and centrifuged, and the resulting supernatant was precipitated with a fourfold volume of 96% ethanol. The precipitate was washed twice with 96% ethanol and dissolved with 3 mL of H_2_O. The resulting solution was used to determine the molecular weight of soluble polysaccharides and the content of GalA by reaction of the sample with 3,5-dimethylphenol in the presence of concentrated H_2_SO_4_ [51]. The alcohol supernatant was used to determine the total amount of sugars using the phenol-sulfur method. The content of PCs was determined in the incubation medium as described above.

### 4.6. EMG and Acceptability Test

Sixteen volunteers (8 men and 8 women) with an average age of 36.5 participated in the study. All of the volunteers were free of masticatory or swallowing dysfunctions and did not have dentures. The experiment was conducted individually. Participants took approximately five minutes to accommodate the experimental environment. Each participant sat on a chair in a vertical but comfortable position with his head in a natural orientation. Participants were asked to rinse their mouths carefully with water between testing gel samples. The subjects were allowed to talk, rinse their mouths, and drink water freely between each trial. Two sessions were held per subject: (1) In the first session, participants scored likeness for A, A-FP0.4, and A-FP1.6 gel samples in an acceptability test (see below). (2) Deliberate unilateral chewing of the gels on the preferred chewing side with EMG recording conducted as reported previously [52,53].

Acceptability was evaluated by the same panelists using a 9-point hedonic scale, where 1 and 9 points are “extremely disliked” and “extremely liked”, respectively [54]. The four samples: A gel (a training sample; it was not taken into account), A, A-FP0.4, and A-FP1.6 gel were presented subsequently. Panelists tasted samples in individual booths under standard light exposure and temperature (20 °C).

### 4.7. Statistics

The significance of the differences among the means in the content of PCs, mechanical parameters, and digestion studies was estimated with the one-way analysis of variance and Fisher’s least significant difference post hoc test. A pared t-test for two dependent means was applied to determine statistically significant differences in EMG variables for different gels. Results from the liking test were analyzed using the Wilcoxon signed-rank test. Statistical differences with a *p*-value lower than 0.05 were considered significant.

## 5. Conclusions

In this study, FP possessing antioxidant activity was efficiently incorporated into the agar gel. The gel structure of A-FP gels was weakened compared to A gel. FP, including polysaccharide chains and their associated PCs, was released from the agar gel after sequential oral in vivo and in vitro gastrointestinal digestion. Here, PCs are released mainly into the simulated colonic fluid. Therefore, A-FP gel can be considered as a promising food gel for enhancing antioxidant protection in the colon. Chewing to readiness for swallowing A-FP gel required less time and fewer chews with less masseter and temporalis activity. Therefore, A-FP gel could be recommended for children, the elderly, patients with dysphagia, and other people who have difficulty chewing hard foods. The A-FP1.6 gel is the softest and carries the highest antioxidant potential because of PCs. However, its lower taste and consistency ratings require further improvement in gel formulation to increase its acceptability for consumers.

Thus, the advantages of agar gel enriched with FP compared to a single agar gel are demonstrated, since pectin provides its biological activity, and agar provides the necessary structural and mechanical properties. Incorporation of pectin into a gelling material, such as agar, can be a promising approach to develop a new food gel with antioxidant properties.

## Figures and Tables

**Table 1:** Characterization of agar gel (A) and agar gels enriched with fireweed pectin (A-FP).

	A	A-FP0.1	A-FP0.4	A-FP1.6
Total PC, μgGAE/g (*n* = 3)	-	23 ± 5 ^a^	84 ± 17 ^b^	418 ± 101 ^c^
Loading efficiency, % (n = 3)	-	86 ± 18 ^a^	78 ± 15 ^a^	97 ± 23 ^a^
*Puncture test:* Hardness, N (n = 12) Consistency, mJ (n = 12)	1.57 ± 0.09 ^a^ 3.18 ± 0.10 ^a^	1.43 ± 0.33 ^a,b^ 2.74 ± 0.55 ^b^	1.35 ± 0.26 ^b^ 2.66 ± 0.45 ^b^	0.91 ± 0.14 ^c^ 1.99 ± 0.29 ^c^
pH (n = 3)	6.22 ± 0.17 ^a^	5.45 ± 0.07 ^b^	5.16 ± 0.03 ^c^	4.77 ± 0.02 ^d^
WL, % (n = 8)	92.2 ± 0.1 ^a^	92.0 ± 0.1 ^b^	91.6 ± 0.1 ^c^	90.3 ± 0.2 ^d^
Serum release, % (n = 12)	0.68 ± 0.08 ^a^	0.67 ± 0.27 ^a^	0.46 ± 0.19 ^b^	0.33 ± 0.09 ^c^
Tg, °C (n = 8)	39.8 ± 1.2 ^a^	40.6 ± 1.6 ^a^	40.1 ± 0.9 ^a^	41.0 ± 0.8 ^b^
Tm, °C (n = 8)	69.4 ± 4.3 ^a^	69.4 ± 4.4 ^a^	70.2 ± 2.6 ^a^	71.0 ± 2.6 ^a^

Mean ± SD. Different letters among gels—*p* < 0.05.

**Table 2:** The texture profile analysis (TPA) properties of A and A-FP enriched gels.

	A	A-FP0.1	A-FP0.4	A-FP1.6
Hardness, N	12.3 ± 0.3 ^a^	10.7 ± 2.8 ^a,b^	10.1 ± 2.0 ^b^	7.3 ± 1.0 ^c^
Cohesiveness	0.43 ± 0.02 ^a^	0.33 ± 0.14 ^b^	0.27 ± 0.12 ^b^	0.20 ± 0.02 ^c^
Chewiness	4.5 ± 0.4 ^a^	3.5 ± 2.2 ^a,b^	3.1 ± 1.8 ^b^	1.5 ± 0.3 ^c^
Springiness	0.83 ± 0.01 ^a^	0.90 ± 0.06 ^b^	0.91 ± 0.06 ^b^	1.03 ± 0.5 ^c^
Resilience	0.16 ± 0.02 ^a^	0.27 ± 0.11 ^b^	0.29 ± 0.12 ^b^	0.45 ± 0.05 ^c^

Mean ± SD. Different letters among gels—*p* < 0.05 (*n* = 12).

**Table 3:** Summary of power law parameters (0.01 < 𝜔 < 20.00 or at 0.1/10.5 Hz).

Gels	Storage Modulus			Viscosity	
	A (Pa)	B (Slope)	R^2^	η_app_ 0.045, Hz	η_app_ 10.500, Hz
A	8550.0	0.054	0.951	12939.6 ± 3048.5 ^a^	152.1 ± 21.9 ^a^
A-FP0.1	9110.6	0.032	0.998	14143.6 ± 3235.0 ^a^	149.8 ± 29.4 ^a^
A-FP0.4	9478.9	0.037	0.996	14574.9 ± 3323.2 ^a^	149.2 ± 29.3 ^a^
A-FP1.6	7646.3	0.047	0.997	11439.0 ± 1918.9 ^a^	129.3 ± 18.7 ^b^

Mean ± SD. Different letters among gels—*p* < 0.05 (*n* = 8).

**Table 4:** Oral processing parameters for chewing of A and A-FP gels.

	A	A-FP0.4	A-FP1.6
Chewing time, s	13.2 ± 8.2 ^a^	12.6 ± 8.7 ^a^	9.3 ± 5.1 ^b^
Number of chews, times	18.3 ± 6.9 ^a^	18.7 ± 12.0 ^a^	13.3 ± 5.9 ^b^
RM activity per sequence, mV × s	0.26 ± 0.08 ^a^	0.25 ± 0.14 ^a^	0.18 ± 0.93 ^b^
RT activity per sequence, mV × s	0.37 ± 0.20 ^a^	0.35 ± 0.20 ^a^	0.19 ± 0.12 ^b^
Total muscle activities per sequence, mV × s	0.63 ± 0.26 ^a^	0.59 ± 0.29 ^a^	0.40 ± 0.21 ^b^
Total muscle activities per chew, mV × s	0.04 ± 0.01 ^a^	0.03 ± 0.02 ^a^	0.03 ± 0.01 ^b^

Mean ± SD. Different letters among gels—*p* < 0.05 (n = 16). RM and RT—right masseter and temporalis muscles.

**Table 5:** Chemical characteristics of polysaccharides used.

Sample	UA ^a^	Gal ^a^	Xyl ^a^	Glc ^a^	Rha ^a^	Ara ^a^	M_w_, kDa
A	3.9	20.0	3.0	0.9	0.2	0.7	280
FP	66.1	1.7	0.9	0.5	1.2	0.9	232

^a^ Data were calculated as wt%. UA—uronic acids, Gal—galactose, Xyl—xylose, Glc—glucose, Rha—rhamnose, Ara—arabinose.

## Data Availability

The data that support the findings of this study are available from the corresponding author upon reasonable request.
